# Comparative study of clinical methods versus ultrasound methods for accurate gestational age determination in different trimesters of pregnancy, Ndop District Hospital, North West region, Cameroon

**DOI:** 10.11604/pamj.2020.37.4.17981

**Published:** 2020-09-02

**Authors:** Prosper Bisahnyui, Claude Ngwayu Nkfusai, Fala Bede, Moraline Kemjei, Catherine Atuhaire, Kenneth Nchanji, Florence Manjong Titu, Samuel Nambile Cumber

**Affiliations:** 1 Institute of Health and Biomedical Sciences, Saint Louis University, Douala, Cameroon,; 2 Center for Global Health Practice and Impact (CGHPI), TIDE-Cameroon Program, Georgetown University's, Yaounde, Cameroon,; 3 Department of Public Health, School of Nursing and Public Health, University of Kwa-Zulu Natal, Durban, South Africa,; 4 Center for Global Health Practice and Impact (CGHPI), TIDE-Cameroon Program, Georgetown University´s, Bertoua, Cameroon,; 5 Faculty of Medicine, Department of Community Health, Mbarara University of Science and Technology, Mbarara, Uganda,; 6 Centre for Health Systems Research and Development, University of the Free State, Bloemfontein, South Africa,; 7 Faculty of Health Sciences, University of the Free State, Bloemfontein, South Africa,; 8 School of Health Systems and Public Health, Faculty of Health Sciences, University of Pretoria, Pretoria, South Africa,; 9 Institute of Health and Care Sciences, The Sahlgrenska Academy, University of Gothenburg, Gothenburg, Sweden

**Keywords:** Comparative, clinical method, ultrasound methods, gestational age, different trimesters of pregnancy, Ndop District Hospital

## Abstract

**Introduction:**

gestational age is the estimated age of gestation from a fetus during its development and this is very important for the mother who wants to know when to expect the birth of her baby and for the health care provider so they can chose the time at which to perform various assessment. However, from the information outlined in this research, it can be seen that last menstrual period (LMP) and follicle-stimulating hormone (FSH) are used to assess gestational age. While GSD, CRL, BPD, HC, AC and FL are biometric parameters that can be measured on a fetus in order to estimate gestational age. Many clinicians and ultrasonologists feel that if they are unable to obtain an accurate measurement at the time, they have sometimes failed to do an adequate job.

**Methods:**

the study was a comparative study on clinical method versus ultrasound method for accurate gestational age determination and also to determine the significance of fetal biometric parameter in GA determination. The study was an observational, cross sectional and participatory study for a period of 5 weeks from the 22^nd^ of January to the 22^nd^ of February 2018. A total of 72(74.2%) ANC cases were sampled during ultrasonography. Gestational ages from their clinic card were recorded. Twenty five questionnaires were given out to 25(26.8%) health care personnel to assess clinical method. Data was analyzed using SPSS version 16 plus and Microsoft Excel 2010.

**Results:**

results showed the best clinical method used was LMP with 9(36%) but when compared to ultrasound, ultrasound presented with greater accuracy of 15(60%) and clinical method 10(40%).

**Conclusion:**

based on the findings, it could be concluded that compared to the physical examination and clinical methods, ultrasound examination of the fetus provided the physician and health care professionals with greater accuracy for gestational age.

## Introduction

A human development is a continuous process that begins when an oocyte (ovum) from the female is fertilized by a sperm (spermatozoon) from a male. Cell division, programmed cell death, differentiation, growth and cell rearrangement transform the fertilized oocyte into a multicellular adult human being. The most developmental changes occur during the embryonic and early fetal periods. The human development divided into prenatal (before birth) and postnatal (after birth) period. There are many changes that occur from the 3^rd^ to 8^th^ week (calculated from the date of fertilization) called as embryonic development [1], it is during this embryonic period that the pregnancy can be first diagnosed by endo-vaginal sonography because of its close proximity to the uterus and the gestational sac, yolk sac diameter, crown rump length can be measured to assess first trimester gestational age [1]. Changes occurring from the 9^th^ week to birth are meaningful because it signifies that the embryo has developed into a recognizable human being called a fetus. For calculating gestational age from last menstrual period (LMP), we need to add two weeks [2]. In fetal life; the skull is developed from mesenchymal connective tissue. The primary areas are two frontal eminences, two parietal eminences, the occipital bone and chondrocranium. The thalamus is an important landmark in fetal sonography. It is the centre of the cranium and is crossed transversely by the biparietal diameter. Sonographically, the two halves of thalamus appear hypoechoic relative to cerebral cortex. Clinically, the gestational periods divided into three trimesters, each lasting three months. At the end of the first trimester, all the major systems are developed.

In the second trimester, the fetus grows sufficiently in size, so that good anatomic detail can be visualized during ultrasonography. At the beginning of the second trimester, the abdominal organs have attained their adult position: the liver, stomach and kidney can be identified. The large bowel is better seen in the third trimester. By the beginning of the third trimester, the fetus can survive if born premature. The fetus reaches a major developmental landmark, like 2.5kg of weight, at 35 weeks of gestation [2]. That is why we can measure the head circumference, biparietal diameter (BPD), femur length (FL), abdominal circumference (AC) during pregnancy. The advent of ultrasound has allowed a more direct means of assessing fetal structures and development of various organs. In the past, gestational age has been established by a combination of the historical information and physical examination. Predictions were passed based on menstrual history index, maternal sensation of fetal movement, assessment of uterine size by bi-manual examination in the first trimester, initial detection of fetal heart tones by Doppler and uterine fundal height measurement [3]. However, it has been reported that, even in best known cases, the menstrual history index and fundal height measurement techniques are also fraught with error.

Timed ovulation and in vitro fertilization with known date of conception are expected to estimate the gestational age accurately. However, in most pregnancies the date of ovulation or conception cannot be as accurately predicted as outlined by other methods and hence gestational age must be estimated by other methods [4]. In limited number of cases, basal body temperature and luteinizing hormone surge indicator are also used for estimating the gestational age with an accuracy of +1- 6 days. The improvement in the field of ultrasonic imaging has made it a strong argument for its use in obstetrics and gynecology. Presently obstetricians and gynecologist are frequently called upon to perform ultrasound examination of the fetus in the first trimester. Compared to the physical examination, ultrasound examination of the fetus is expected to provide the physician and healthcare professionals with estimates of greater accuracy. For most pregnant women who come for antenatal clinic (ANC), their LMPs are usually very unreliable because most of them have no knowledge of their last LMPs, or history of irregular menstrual cycles or have been on oral contraceptives within two months of their last LMP [5]. Many ultra sonographers feel that if they are unable to obtain an accurate measurement at the time of an ultrasound examination that means they have sometime failed to do an accurate job. Thus, the aim of this study is to carry out a comparative study on clinical method versus ultrasound method for accurate gestational age determination in different trimesters or pregnancy.

## Methods

**Materials:** questionnaires, echography unit with specifications: diagnostic ultrasound equipment: model: BU-907; power: 100V; P/S: 100-240V 50/60Hz; serial no: 1009060099; manufactured by Biocare Medical Systems Corporation with a Convex; transabdominal probe 3.75MHz; echography gel, disposable tissue.

**Study design:** this study was an observational, participative, cross sectional and descriptive study.

**Study setting and area:** the research was carried out in Ndop District Hospital in the North West Region of Cameroon. Ndop is a Sub-Division of Ngoketunjia Division. It has a suitable population estimated above 12 thousand inhabitants, which is suitable for this research.

**Study population:** all pregnant women who attended ANC at Ndop District Hospital constituted the research population; health care providers in the hospital who attended to pregnant women during this period.

### Selection criteria

**Inclusion criteria:** ANC women who come for ultrasound and know their last menstrual period. Of these, 72 pregnant women who had their clinic cards and 25 health personnel who signed the consent form participated in the study; all concerned healthcare givers who attended to pregnant women during the time of the study and gave consent were included in the study.

**Exclusion criteria:** all women without an LMP and ANC card were excluded; women with twin gestations and malformations were excluded; healthcare givers who did not give their consent were excluded.

**Study duration:** the study lasted for a period of five weeks beginning from the 22^nd^ of January to 22^nd^ of February 2018.

### Sampling

**Sample size calculation:**

$$\text{Emax}=\frac{z\alpha }{2}\left\{\sqrt{\text{p}}\left(1-\text{p}\right)/\sqrt{\text{n}}\right\}$$

Where Emax = max error; Zα/2=level of confidence; p = prevalence; n = sample size;

$$\frac{E\max}{\frac{z\alpha }{2}}=\sqrt{\text{p}}\left(1-\text{p}\right)/\sqrt{\text{n}}$$

$$\sqrt{\text{n}}=\frac{\frac{z\alpha }{2}\left(\sqrt{\text{p}}\left(1-\text{p}\right)\right)}{\text{Emax}}$$

and squaring both sides,

$$\text{n=}\left(\frac{\frac{\text{z}\alpha }{2}}{\text{Emax}}\right)\text{p}\left(1-\text{p}\right)$$

using p= 6.5% (0.065), gotten from the literature review and Zα/2=1.96, got from the standard table, working at 95% confidence and Emax=5% (0.05) n=(1.96/0.05)^2^0.065(1-0.065); n=99.88. Therefore n=100 patients/participants to 3 significant figures but due to the short duration of the study, only 97 participants were registered.

**Random or probability sampling method:** simple random sampling method was used such that each participant had equal chance of participating.

**Data collection procedures and analysis:** the ultrasonographic biometric parameters of gestational sac diameter, CRL, BPD, abdominal circumference, femoral length, ANC cards, as well as LMPs were used to collect and analyze the data.

**Data management and data analysis:** the data was saved on note books, USB drives and analyzed using a statistical software analysis package SPSS version 19.

**Ethical considerations:** for ethical consideration, clearance was obtained from the medical diagnostic imaging department. Patient´s consent, as well as authorization from medical committees of the hospital and public health delegation of health in the North West region.

**Study limitations:** some problems encountered were: delay in obtaining clearance from the public health delegation of the North West region. Lack of sensitization by nurses during ANC on the importance of ultrasound check up during pregnancy. Most ultrasound unites do not make use of basic parameters such as HC, AC etc. which was a limitation as the unit I used dint make use of the two parameters mentioned above.

## Results

**Demographic characteristics of the study population:** the highest number of participants was females 80(82.5%) as compared to males 17(17.5%) with the highest age group being 20-29 years 40(41.2%) while the lowest were 40 and above 18(18.6%). Among the participants, 72(72.2%) were pregnant women while 25 were health personnel which included 4 (4.1%), medical doctors 20(20.6%) nurses and 1(1%) midwife (Table 1).

**Table 1:** demographic characteristics of the study population

Variable	Frequency	Percentage (%)
Gender: male	17	17.5
Female	80	82.5
Total	97	100
Age group: 20-29 years	40	41.2
30-39 years	39	40.2
40 years+	18	18.6
Total	97	100
Occupation: medical doctor	4	4.1
Nurse	20	20.6
Midwife	1	1
Pregnant women	72	74.2
Total	97	100

**Figure 1:**
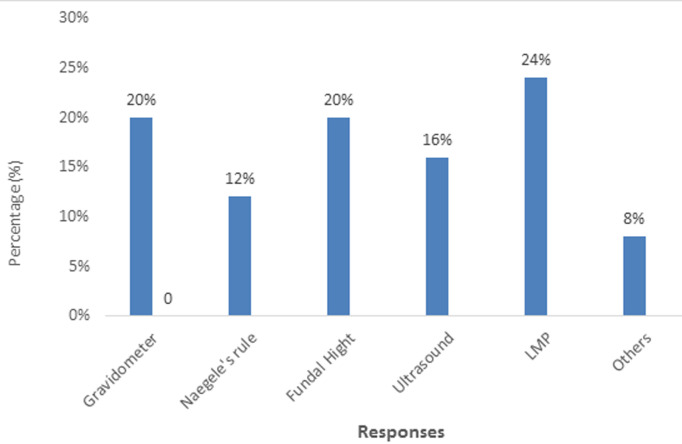
percentage distribution of various methods used to determine gestational age generally by the 25 health personnel

**Percentage distribution of various methods used to determine gestational age generally by the 25 health personnel:** the methods used by the clinicians in determining gestational age with the most used being LMP with a percentage of 24 and the least method included other methods for example quickening which represented 8% (Figure 1).

**Various methods used in different trimesters to determine gestational ages:** the various methods used by clinicians in various trimesters to determine gestational age, in which LMP was the most used method 10(40%) in the first trimester but as it proceeded to the second trimester symphysis-fundal height (SFH) measurement took over being the most used method 10(40%) and 12(48%) respectively (Table 2).

**Table 2:** the various methods used in different trimesters to determine gestational ages

	Various Trimesters			
Methods Used	First Trimester	Second Trimester	Third Trimester	Total
Gravidometer	6(24%)	3(12%)	3(12%)	12
Naegele's rule	4(16%)	3(12%)	2(8%)	9
Fundal height	2(8%)	10(40%)	12(48%)	24
Ultrasound	2(8%)	4(16%)	3(12%)	9
LMP	10(40%)	4(16%)	4(16%)	18
Others	1(4%)	1(4%)	1(4%)	3
Total	25(100%)	25(100%)	25(100%)	75

**Figure 2:**
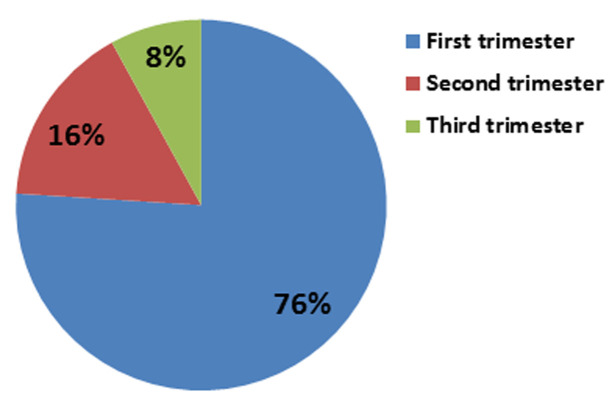
percentage distribution of respondents based on their opinion about the best trimester to determine gestational age

**Percentage distribution of respondents based on their opinion about the best trimester to determine gestational age:** the percentage distribution of respondents based on their opinion about the best trimester to determine gestational age where 76% of respondents said the first trimester is the best period to determine gestational age, 4(16%) choose the second semester as the best period and 8% said the third trimester is the best period in determining gestational age (Figure 2).

**Distribution of respondents with respect to their choice on the best clinical method for determining gestational age:** the distribution of respondent with respect to their choice of the best clinical method for determining GA, in which the best method chosen was LMP 9(36%) second by SFH 7(28%) and the least being other methods such as quickening (Table 3).

**Table 3:** distribution of respondents with respect to their choice on the best clinical method for determining gestational age

Variable	Frequency	Percentage
Gravidometer	4	16%
Fundal height	7	28%
Naegele's rule	4	16%
LMP	9	36%
Others	1	4%
Total	25	100%

**Percentage distribution of respondents based on their preference between ultrasound and the best clinical method used in determining gestational age:** a percentage distribution of respondent based on their preference between ultrasound and the best clinical method used in determining GA, in which 15(60%) preferred ultrasound for accurate GA determination while 10(40%) said clinical method was preferable.

**Figure 3:**
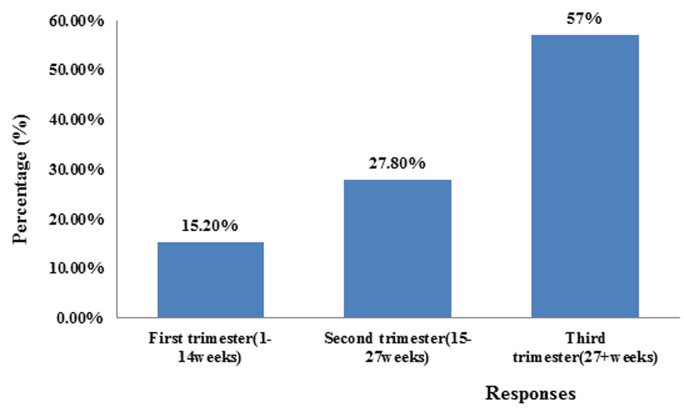
percentage distribution of pregnant women according to the trimester they were scanned

**Percentage distribution of pregnant women according to the trimester they were scanned:** the percentage distribution of pregnant women according to the trimester they were scanned, the highest number of women was scanned in the third 41(57%) trimester while the least number of women were scanned in the first trimester of pregnancy 11(15.2%) (Figure 3).

**Figure 4:**
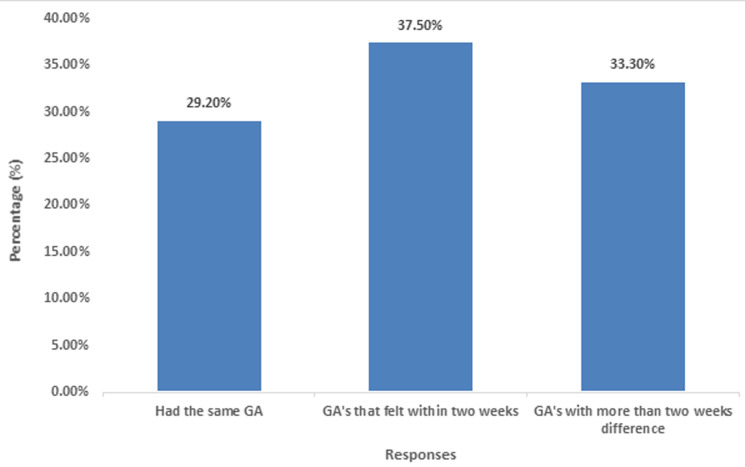
showing distribution of gestational ages with respect to variation between ultrasound and clinical method

**Distribution of gestational ages with respect to variation between ultrasound and clinical method:** the distribution of gestational ages with respect to variations between ultrasound and clinical method, 21(29%) of respondents had same GA, 27(33%) felt within two weeks of accuracy and 24(38%) had a difference of more than or less than two weeks when ultrasound GA was compared to the clinically determined ages written in their clinic cards (Figure 4).

## Discussion

The highest number of participants were female (82.5%), because, the greater sample constituted pregnant women and most nurse and midwives are women, too, while 17.5% were male, due to the fact that health personnel like, doctors and some nurses who were males, were included in the study which constituted a smaller portion of the study. The highest age being 20-29 year (40.2%). The reason being that this age group is good for child bearing, as the minority age group was 40 years and above (18.6%) with reason being that most women get into menopause after the age of 45, but since the study included, health personnel´s, they were most of the nurses at ages of 40 and above, which was an advantage to the study because of their clinical experience on gestational age assignment. The questionnaires were give to 4(4.1%) medical doctors, 1(1%) midwife and 20(20.6%) nurses, reason being that many midwives have not been trained and sent to the field to practice, so the nurses performed a double function and the clinic cards of 72(74.2%) pregnant women were accessed, after ANC. The percentage distribution of various methods used to determine gestational ages, generally by health personnel, majority of them 6(24%) said LMP was the best method used in determining gestational age, because this estimation assumes that conception occurs on day 14 of the cycle. The fallacy in this assumption is that the menstrual cycle varies from individual to individual and basing GA, on the LMP tends to result in an over estimation [6]. While the least method used was 2(8%) of other methods, such as, period of quickening, relating of LMP to an event, or asking husband of the woman. These methods were less accurate, because it leads to over or under estimation of gestational age.

This is in line with a study carried out by Deputy *et al*. (2017) in which he analyzed 912 women with ultrasound and prospectively collected LMP estimate of GA, LMP and ultrasound were moderately correlated, though GA was slightly over estimated by LMP, (median 273.9 days) and more greatly over estimated by Farr´s examination (median of 286 days). In the first trimester, the most used method is the LMP method 10(40%). This is most preferable by health personnel, because, the LMP is easy to remember, but in the second and third trimester, the most used method is the fundal height measurement because in the rural areas, women come sometimes to ANC late in the second trimester and the LMP cannot be recalled thus the fundal height method is preferred. This goes in line with an article published by [7] on the best method to accurately determine gestational age in rural Guatemala which remains a problem to researchers, LMP and SFH, for 117 was used to determine which method provided the estimate of GA using Uss as reference. LMP estimated GA, explained 46% of the variance in GA estimated by Uss whereas neonatal examination, explained only 20%. Thus it was concluded that LMP provided the best estimate of GA, while SFH measured during 2^nd^trimester may provide a reasonable alternative when LMP is unavailable. The percentage distribution of respondents, based on their opinion about the best trimester to determine gestational age 76% which is first trimester which the majority of the participants choose as the best period to determine GA whereas, 8%, only choose third trimester.

This is very true because the LMPs can be easily recalled in the first trimester unlike the second and third as many weeks have passed and many activities taken place and the women in this rural area could barely remember their LMPs. This is also in line with article no. 303 publishes February 2014 by the Canadian Society of Obstetricians. Which showed that CRL was very accurate at 17 weeks and similar to biparietal and the CRL should be used up to 84mm, because of its accuracy. Thus, the best parameter too by USS is found in the first trimester percentage distribution of respondents based on their preference between ultrasound and the best clinical method used in determining gestational age. After assessing the method and best clinical method of assessing clinical age, the method was compared to ultrasound and 60% of them preferred ultrasound whereas 40% preferred clinical method. This is because, in the rural areas women sometimes start the ANC late in the second trimester and to the limitations indicated by LMP and SFH height above, the best option is to send the women for USS. Meanwhile, some experienced clinicians still held on to the clinical methods for GA determination, example, in a comparative study carried out by L Geerts *et al*. in South Africa in which a total of 1342 pregnancies were analyzed.

The accuracy of determining was similar to certain and uncertain LMP, SFH was less accurate but US-based dating was accurate with 85% prediction within 14 days. USS reduced the number of assumed pre/post term delivery. The distribution of respondents with respect to the choice on the best clinical method for determining gestational age. Again, from past literature review such as the study carried out by [8] LMP stands out as the most accurate. Though with its own complications which occur when women did not know of LMP or gave wrong LMP which led to over or underestimation of GA. SFH measurement was less accurate, this backed by the study carried out by [9], who published an article in the Journal of the Royal Society Interface, on the estimation of gestational age from SFH measurement for poor setting in Thailand in which there was a large variation between the measurement and variation with individuals, each mother had a different growth pattern for SFH, versus gestational age, this was due to certain facts discovered in the field such as, twin pregnancy, fibroids in pregnancy, full bladder.

This was as a result of the fact that many pregnant women in the rural areas did not start ANC early in their pregnancy and which qualifies the study carried out by [9] in which, there was much variation in the SFH measurements and only during this period will the sonographer have to use more than two, or two parameters to come out with accurate measurements [10]. Since LMP is mostly forgotten and SFH presented with a lot of problems. From past literature review, LMP has an advantage over the other measurements, like SFH and fars estimate [11], though it has its own limitations, SFH height measurement which was also used in most rural area like rural Thailand and rural Guatemala [12]. White *et al*. also led to over estimation due to some reasons like uterine fibroids, racial variations, but was used when ultrasound was not available but dating back to the late 1970s with the advent of ultrasound and use of multiple biometric parameters, ultrasound continues to present with more accuracy when compared to clinical methods. This is in line with the study carried out by [13,14], department of obstetrics and gynecology, South Africa in which 1342 pregnancies were analyzed and USS presented with an accuracy of 85%. Thus, these factors led to the variations which occurred, accounted for 33% great difference in GA between ultrasound and clinical method.

## Conclusion

Based on the above findings, it could be concluded that compared to the physical examination and clinical methods, ultrasound examination of the fetus provided the physician and health care professionals with greater accuracy for gestational age. Thus GA assessment is important and provide a more accurate dating than the menstrual dating which improves antepartum and postpartum management.

**Recommendation:** nurses and midwives need to be sensitized on the importance of counseling pregnant women for routine ultrasound checkup at least two times per pregnancy. Ultrasound operators need to follow guidelines and standards when scanning pregnant cases to provide accurate information to clinicians and other health personnel´s for follow up.

### What is known about this topic

- Ultrasound determination of menstrual age;
- A comparison of pregnancy dating methods commonly used;
- Evaluation of gestational age from menstrual age.

### What this study adds

- Comparison to the physical examination and clinical methods, ultrasound examination of the fetus provided the physician and health care professionals with greater accuracy for gestational age;
- GA assessment is important and provide a more accurate dating than the menstrual dating which improves antepartum and postpartum management.
